# Health behaviours and beliefs in individuals with familial pancreatic cancer

**DOI:** 10.1007/s10689-019-00143-7

**Published:** 2019-09-14

**Authors:** Meghan Underhill-Blazey, Traci Blonquist, Janette Lawrence, Fangxin Hong, Matthew B. Yurgelun, Sapna Syngal

**Affiliations:** 1 Dana-Farber Cancer Institute, 450 Brookline Avenue, LW 522, Boston, MA 02215 USA; 2 Massachusetts General Hospital, 55 Fruit Street, Boston, MA 02114 USA; 3 Brigham and Women’s Hospital, 75 Francis Street, Boston, MA 02215 USA

**Keywords:** Pancreatic cancer risk, Surveillance, Health behaviour, Patient reported outcomes

## Abstract

Individuals at high risk for pancreatic cancer are recommended surveillance and healthy lifestyle behaviours and patient experience with recommendations are understudied. To describe engagement and experience with surveillance, tobacco and alcohol use, health beliefs and motivation (Champion Health Belief Measure) and the relationship with personal, psychosocial (Impact of Event Scale), and familial characteristics. Interest in integrative therapies (complementary therapies) are described. A multi-site cross-sectional survey including individuals at high risk for pancreatic cancer with no diagnosis of pancreatic cancer who have been evaluated at a comprehensive cancer center. Descriptive statistics and Wilcoxon rank sum test and Fisher’s exact test were used to assess univariate associations. Of the 132 respondents (72% response rate), 92 (70%) reported undergoing surveillance which was associated with older age (p = 0.001). Of which, 36% and 51% report that magnetic resonance imaging (MRI) or endoscopic ultrasound (EUS), respectively, were uncomfortable; 22% and 30% dread the next MRI or EUS, respectively. Of those who reported alcohol consumption (n = 88); 15% consumed 1 or more drinks daily and no alcohol consumption was associated with higher Impact of Event scale scores (p = 0.024). A total of six participants were currently smoking every day or some days. Participants reported high motivation to engage in heathy behaviours and 92% were interested in integrative therapies. In these select participants, most were engaging in pancreatic cancer surveillance, alcohol intake was moderate, and tobacco intake was minimal. Modifiable factors, such as experience and comfort with surveillance could be addressed. The sample is motivated to engage in behavioural health intervention.

## Introduction

### Background/rationale

Pancreatic cancer is projected to increase from the fourth to the second leading cause of cancer-related deaths by 2030 [1]. Poor outcomes are, in part, due to the few signs and symptoms of pancreatic cancer and the late stage of diagnosis. Only a minority of patients are diagnosed with the earliest stage of pancreatic cancer, when surgical cure is most likely [2].

Individuals with risk related to familial or hereditary factors face up to a 3–8-fold increase in risk for developing pancreatic cancer compared to the general population risk of 1.5%, with the highest risk group having known genetic syndromes in which pancreatic cancer is a component [3]. Individuals are most commonly identified as having familial pancreatic cancer risk due to having two or more relatives affected with pancreatic cancer [2, 3].

Those at highest risk, or those with a relative risk greater than fivefold and unaffected with cancer, are currently offered cancer surveillance with endoscopic ultrasound (EUS) which requires sedation, and/or magnetic resonance imaging (MRI) annually starting at age 50 or at 10 years prior to the earliest diagnosis of pancreatic cancer in the family [3]. If surveillance identifies a suspicious solid or cystic lesion or intraductal papillary mucinous neoplasm (IPMN), the follow-up interval may be shortened or surgical resection may be recommended [3]. Current data suggest that up to 39% of patients will have an identified abnormality that warrants further evaluation [4, 5].

Modifiable behaviours such as current tobacco use, and heavy alcohol consumption are related to a significantly increased relative risk for pancreatic cancer. Therefore, in addition to engaging in surveillance, at-risk individuals are encouraged to maintain healthy lifestyle behaviours, including minimal alcohol intake and avoidance of tobacco [3]. Modifying health behaviours could reduce risk for pancreatic cancer, and also reduce risk for other co-morbid conditions, such as heart disease or diabetes mellitus, or cancers, such as breast cancer, colon cancer, and lung cancer, a cascade benefit of lifestyle modification for pancreatic cancer risk [6].

Individuals living with inherited pancreatic risk may face cancer related worry or psychosocial distress associated with risk and family experience [7, 8]. Integrative therapies (also known as complementary therapies), such as meditation, yoga, or exercise, have been found to reduce feelings of distress and contribute to overall well-being, and are recommended for the care of individuals with cancer [9]. There are limited data related to the perceptions of at risk individuals on integrative therapies, however, these therapies are promising for providing supportive care and further exploration is warranted.

### Objectives

To describe engagement in surveillance, experiences with surveillance, tobacco use, alcohol use, health beliefs and health motivation and the relationship with personal, psychosocial, and familial characteristics. Engagement in and interest in integrative therapies are also described. The analysis presented here is part of a larger study aimed at describing relationships between personal and demographic factors and psychosocial and health behaviour outcomes in individuals with high pancreatic cancer risk. Personal characteristics associated with psychosocial outcomes have been previously reported [7].

## Methods

### Study design

Institutional Review Board approval was obtained from the lead study site. We conducted a multi-site cross-sectional descriptive survey study using a tailored mailed survey method [10]. Surveys were available for completion in paper or online.

### Study participants and setting

Participants were identified from two academic medical centers in the northeastern United States. Eligible participants were English speaking adults with no history of pancreatic cancer who met criteria for inherited or familial pancreatic cancer risk [3] and had been recommended by a physician to undergo pancreatic cancer surveillance. Participants were first sent a mailed information sheet along with the survey, with information pertaining to consent and how to complete the survey either by paper or online. Participants were contacted by mail two more times for recruitment. A $5 incentive was provided to all participants in the first mailing.

### Variables and measurement

Preliminary qualitative research conducted by the study team [8] and the Health Belief Model [11] guided the identification of survey items. All measures were self-reported by the participant.

#### Demographics

Information pertaining to self-reported *personal cancer history*, *age, marital status, experience caring for a person with cancer* (*yes/no*)*, experience with familial cancer death* (*yes/no*) *and length of time since that experience* (*less than or equal 5* *years or 6 or more years ago*) was collected.

#### Surveillance and health behaviours

Participants were asked to report *pancreatic cancer surveillance behaviours* (*endoscopic ultrasound and MRI*) or *behavioural intentions,* and *experiences with surveillance* (*level of comfort with procedure and amount of dread for next procedure* [12])*. Smoking and alcohol use* were measured based on adapted measures from the Health Information National Trends Survey (HINTS) and evaluated based on current American Cancer Society guidelines which recommend tobacco cessation or avoidance and no more than 1–2 alcoholic drinks per day [6]. *Use or interest in using other health therapies, or integrative therapies*, were assessed with 2 study specific items asking participants to select (1) therapies that they had previously used and (2) therapies that they would consider using in the future. Participants could select all that apply from a list of 17 response options. Options included, for example, massage, acupuncture, meditation, yoga, or nutrition consultation.

Health Belief Model variables were measured using the widely used and validated Champion Health Beliefs scale (CHBS) [13], adapted for pancreatic cancer surveillance with permission from the author. The CHBS measures key constructs that predict an action, such as a health behaviour. As posited by the model, susceptibility (e.g. Extremely likely I will get pancreatic cancer in the future) and severity (e.g. the thought of pancreatic cancer scares me) indicate that the participant has recognized a threat, in this case pancreatic cancer, which is necessary to promote health behaviour. Benefits (e.g. when I get pancreatic cancer surveillance I don’t worry as much about pancreatic cancer) and barriers (e.g. having pancreatic cancer surveillance would be painful) are specific to the health behaviour, in this example pancreatic surveillance, and to engage a participant must find high benefit and low barriers to the behaviour. Health motivation (e.g. I exercise at least three times a week) is the individual’s readiness to be concerned about health promotion and is a determinant of engaging in future health behaviours.

Subscales were scored on a range of 1 (strongly disagree) to 5 (strongly agree) with a higher score indicating more of the construct. If 1 item in the subscale was missing, it was replaced by the average of the remaining items. The possible scores for the susceptibility subscale are from 5 to 25, for severity 7–35, and for health motivation 7–35. The benefits subscale (four items, possible scores 4–20) is only asked to those that answered “yes” to engaging in surveillance and the barriers subscale (five items, possible scores 5–25) is only asked to those that answered “no”.

Psychosocial factors: Pancreatic cancer risk related distress was measured by *The Revised Impact of Event Scale* (*IES*) [14], a 22 item measure validated and widely used to measure the outcome of distress associated with hereditary cancer risk and cancer screening. The measure is scored ranging from 0 to 88, with higher score indicating more distress and a score of 24 or greater indicating post-traumatic stress. Cancer Risk Perception was measured using adapted version of items included in the Levy et al. publication measuring perceptions of chance of developing pancreatic cancer and how this risk compares to the average person of the same age [15]. For this analysis, we categorized risk perception into 40% or less or 50% or greater due to current objective risk estimates for pancreatic cancer which indicate that most objective risk for cancer falls below 40% [3].

### Study size and analysis

The study was exploratory and therefore no formal power calculation was conducted. All continuous items and measures were summarized with descriptive statistics (median/range) and compared between groups with the Wilcoxon rank sum test. Categorical items and measured were compared between groups with the Fisher’s exact test. Exact 95% confidence intervals were calculated for screening intent and health behaviours. Due to a small number of participants reported as current smokers, only descriptive data is presented pertaining to smoking.

## Results

### Participant characteristics

Of the 186 individuals sent a survey, 133 responded (72% response rate) and 132 had analyzable data. Complete demographics and psychosocial characteristics of the study sample are reported elsewhere [7]. All participants were adults who did not have a pancreatic cancer diagnosis. Participants had been evaluated in a comprehensive cancer center by a genetic counselor and physician and have been identified as having high risk for pancreatic cancer due to personal or familial factors. In total, 92 (70%) indicated having engaged in surveillance. Personal characteristics of participants in relation to engagement in pancreatic cancer surveillance, of the 128 reporting, are presented in Table 1.

**Table 1:** Engagement in surveillance based on personal characteristics

	Overall		Have you ever had pancreatic cancer surveillance?				p-value^b^
			No		Yes		
	N	%	N	%	N	**%**	
N	128	–	36	–	92	–	–
Gender^a^							0.14
Male	41	32	8	22	33	36	
Female	86	67	28	77	58	63	
Marital status^a^							0.13
Unmarried/partnered	36	28	14	39	22	24	
Married/partnered	91	71	22	61	69	75	
Education level^a^							0.73
≤ High school	10	8	2	6	8	9	
> High school	117	91	34	94	83	90	
Personal history of cancer							0.44
No	67	52	21	58	46	50	
Yes	61	48	15	42	46	50	
Have you taken care of a very ill parent/family member							1.00
No	43	34	12	33	31	34	
Yes	85	66	24	67	61	66	
Have you lost a family member to cancer							0.097
No	7	5	4	11	3	3	
Yes	121	95	32	89	89	97	
Have you been recommended screening							< 0.001
No	20	16	15	42	5	5	
Yes	107	84	20	56	87	95	
Don’t know	1	< 1	1	3	0	0	
Risk perception^a^							0.22
< 50%	50	39	18	50	32	35	
≥ 50%	69	54	17	47	52	56	
IES^a^							0.23
IES < 24	106	83	28	78	78	85	
IES ≥ 24, indicating PTSD	16	13	7	19	9	10	

^a^1 unreported for gender, marital status, and education; 9 for risk perception; 6 for IES

^b^p-value excludes unreported

### Main results

Surveillance behaviours: Of the 92 participants responding “yes” to having surveillance, 91 reported the type of surveillance. Overall, 16 participants (18%) reported undergoing EUS alone, 16 (18%) MRI alone, 57 (63%) both EUS and MRI, and 2 (2%) reported another type of surveillance. Of the 57 that indicated having both MRI and EUS, 55 answered the item about preference; 27 (49%) preferred MRI, 5 (9%) preferred EUS and 20 (36%) did not have a preference (3 responded “other”). For the 92 participants who underwent pancreatic cancer surveillance, 89 answered the question pertaining to a willingness to continue surveillance in the future, and 79 (89%; 95% CI 80–94%) intend to continue. There were no statistically significant demographic factors associated with intent to continue. Table 2 summarizes participants’ experiences and level of comfort with undergoing the procedures. In summary, 36% and 51% report that MRI or EUS were a little to very uncomfortable, and 21% and 30% have some or a lot of dread for the next MRI or EUS procedure, respectively.

**Table 2:** Self-reported experience with endoscopic ultrasound (EUS) and magnetic resonance imaging (MRI)

	Overall			
	MRI (n = 72)		EUS (n = 73)	
	N	%	N	%
Experience				
Very uncomfortable	3	4	10	14
A little uncomfortable	23	32	27	37
Somewhat comfortable	16	22	15	21
Very comfortable	30	42	21	29
Dread next procedure				
Not at all	36	50	34	47
A little	21	29	17	23
Somewhat	8	11	14	19
A lot	7	10	8	11

Alcohol and tobacco use: In total, 130 participants answered the question regarding alcohol consumption. Of those, 42 (32%) reported not drinking any alcoholic drinks per week. Nineteen individuals (15%) reported consuming 7 or more alcoholic drinks per week, 6 of whom (5%) reported 10 or more a week. All others (n = 69) reported consuming between 1 and 6 alcoholic beverages weekly. The proportion of patients that did not drink any alcohol was significantly higher for those with an Impact of Event Scale score indicating post-traumatic stress (greater than or equal to 24, n = 17) compared to those with a score less than 24 (59% vs. 29%; p = 0.024).

Out of the total 132 participants, 40 (30%) indicated smoking 100 or more cigarettes in a lifetime. Of the 40, 39 answered the follow-up question “How often do you now smoke cigarettes?” and 33 (85%) do not smoke cigarettes at all, 3 (8%) smoke some days, and 3 (8%) smoke every day. Of the six participants currently smoking, five are seriously considering quitting in the next 6 months, the remaining one participant did not respond.

Use of other health therapies: 129 participants answered the question regarding use of other health therapies (i.e. integrative therapies) in the past of which 93 (72%) indicated using some form of integrative therapy (for example, massage, acupuncture, medication). For the item addressing future interest in integrative therapies, 117 participants answered the question of which 108 (92%; 95% CI 86–94) indicated being open to using at least one therapy in the future.

**Fig. 1:**
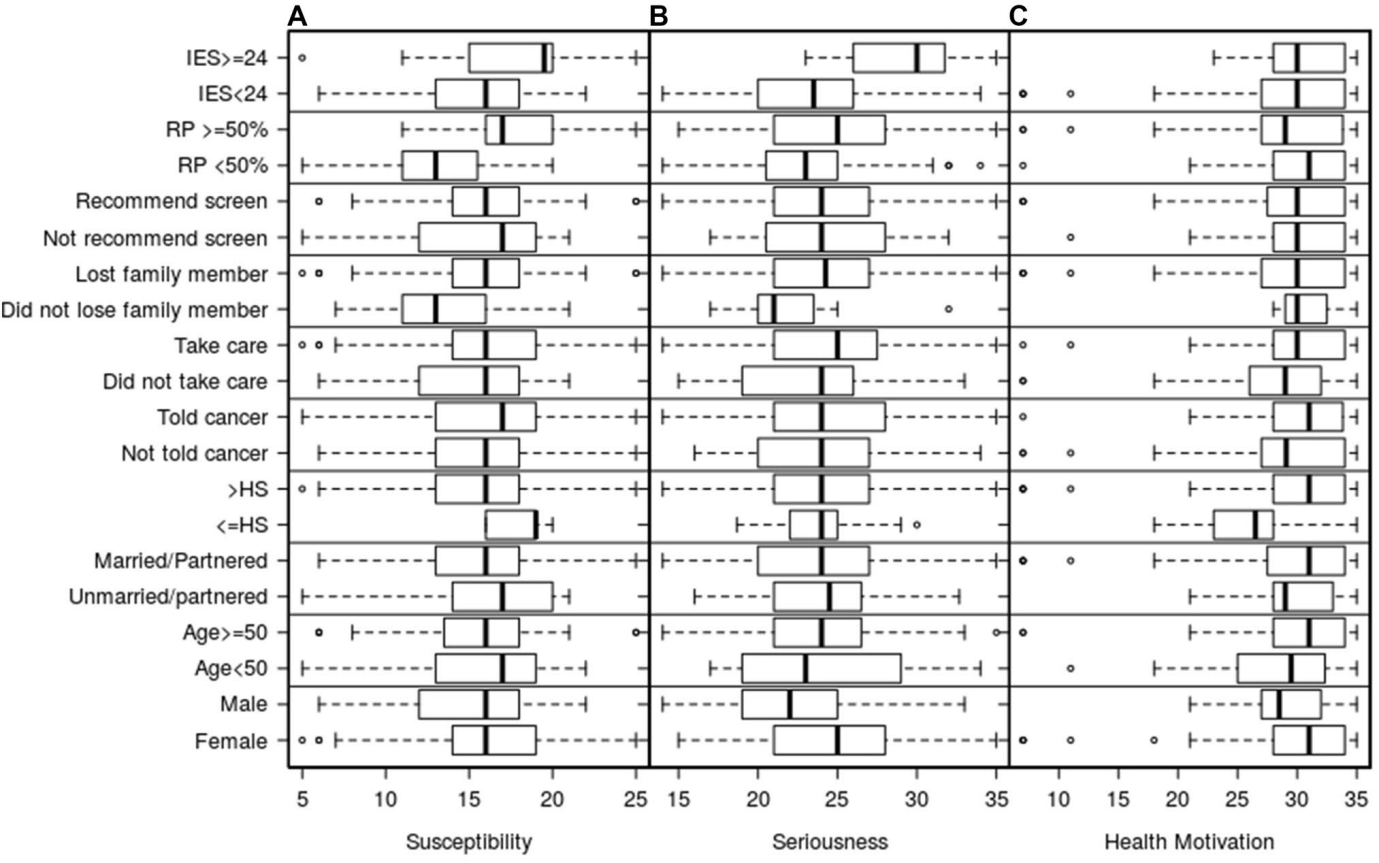
Distribution of susceptibility, seriousness, and health motivation subscale scores by demographic. Note: *IES* impact of event scale, *RP* risk perception; Lost family member/did not lose family member = experience with loss of a family member to pancreatic cancer; Take care/did not take care = role as a caregiver to a family member with cancer; Told cancer/Not told cancer = personal history of a cancer diagnosis; HS = high school

Health beliefs and motivation: Fig. 1 describes the score outcomes for the susceptibility and severity, or perceived threat of pancreatic cancer, and general health motivation subscale scores. The distribution of responses within the susceptibility subscale were significantly different based on risk perception (p < 0.001) and IES Scores (p = 0.026), with those with higher risk perception and higher pancreatic cancer risk related distress perceived more susceptibility. Seriousness subscales scores differed based on gender (p = 0.014) and IES scale scores (p < 0.001) with females and those with more distress perceiving more seriousness. Health motivation, or current perceived engagement in health promotion, significantly differed based on gender (p = 0.015), education level (p = 0.016), and having taken care of an ill parent or family member (p = 0.029), with females, those with greater than a high school education, and having had taken care of a family member reporting higher motivation.

## Discussion

In the present study, we describe health behaviours (pancreatic cancer surveillance and experience, tobacco, alcohol), health beliefs and motivation and the relationship with personal, psychosocial, and familial factors, as well as engagement and interest in integrative therapies in individuals at elevated risk of pancreatic cancer. Overall, most participants engaged in surveillance and intended to continue. A subset did find both surveillance procedures uncomfortable, with EUS being perceived as uncomfortable more frequently compared to MRI. A subset of the sample (4.6%) reported consuming levels of alcohol that may exceed current American Cancer Society recommendations of 1 to 2 alcoholic drinks per day. Additionally, six individuals were current smokers, five of whom were interested in quitting. Overall interest or engagement in integrative therapies was high and health motivation was high (ex. engagement in diet and exercise).

Most participants did not find the surveillance MRI uncomfortable and did not dread the next procedure. Of the two surveillance procedures, EUS resulted in more frequent reports of being uncomfortable and slightly more reports of dreading the next procedure. In a longitudinal study measuring the same outcome of comfort after pancreatic surveillance, Konings et al. (2016) had similar findings. Here the authors report that on average 10–11% of the 140-person sample felt that MRI or EUS was very uncomfortable respectively and found that 4% and 11% dreaded the next procedure at the highest level. Longitudinal findings from the Konings study suggest that after the first EUS, experiences of dread drop to similar levels as found with MRI [12]. In our present study, though some discomfort was reported in this one cross-sectional measurement, most participants intended to continue with surveillance and reported barriers to surveillance are low. These findings suggest participants may benefit from expectation management and support in preparing for the procedure to reduce feelings of discomfort or dread.

Participants reported a moderate perceived susceptibility to pancreatic cancer and that it was a serious disease, therefore indicating that they perceive pancreatic cancer as a threat—a prerequisite to health action. The sample reported high health motivation or readiness to engage, specifically in those with higher education or those who had been a caregiver to an ill family member, with high benefits and few barriers to engaging in pancreatic surveillance. Based on these findings and existing health behaviour theory [16, 17], we find that individuals with high risk for pancreatic cancer may be approachable for health behaviour change intervention.

Additionally, pancreatic cancer risk assessment and consultation, especially for those with heightened risk perception or experience as a caregiver, may be a teachable moment to engage in meaningful behaviour change modification [18]. Our findings further support that participants presenting for pancreatic cancer risk associated care are highly motivated to engage in health behaviour intervention. In part, the reason for this motivation stems from the personal and family experience of living through a loved one’s pancreatic cancer diagnosis [8]. Though the sample was a select group engaging in surveillance, recent data suggests that the field of pancreatic cancer genetics is moving towards recommending germline genetic testing in all individuals with pancreatic cancer [19, 20]. Therefore, soon, there will be a growing number of family members at risk for inherited pancreatic cancer and in whom regular surveillance will have potential health benefits. Therefore, the time of genetic consultation for at risk individuals is an opportune time to promote cancer prevention behaviours and to potentially improve the health of individuals and families impacted by pancreatic cancer.

Previous literature has found that those who seek genetic counseling due to familial cancer risk have similar modifiable cancer risk factors as the general population [21, 22]. In our sample, most individuals were not consuming high levels of alcohol as defined by the American Cancer Society or consuming tobacco. However, a subset report substance use, and current epidemiologic data suggests that heavy alcohol intake of three or more drinks per day and any tobacco exposure can increase risks for developing pancreatic cancer. Tobacco use is particularly concerning and has been noted to lead to a fourfold increase in pancreatic cancer risk in individuals with familial pancreatic cancer and can lead to cancers occurring at a younger age [23, 24].

Data suggest that those presenting for genetic consultation or for high risk cancer care are engaging in modifiable behaviours that may contribute to cancer risk. However, despite this knowledge, consultations with genetic teams infrequently discuss modifiable lifestyle factors [25] and are inadequately addressing health promotion related needs. There is a need to further evaluate how to help patients undergoing genetic counseling and testing engage in healthy behaviours. Improving lifestyle and health behaviours in at risk individuals has the cascade benefit of not only reducing a potential for cancer risk but improving overall health and quality of life. Considered in the context that this sample was highly motivated to engage in healthy behaviours, targeted and tailored health messages, that address tobacco or alcohol as necessary, could be an important future clinical and research consideration.

Our findings suggest that not only is risk and risk perception a catalyst for engaging in health promoting activities, experiences within the family are as well and caring for a seriously ill loved one in the context of inherited pancreatic cancer risk is associated with higher reported health motivation. We previously identified that these factors were also associated with higher psychosocial distress compared to others in the same sample [7]. Therefore, when encountering an individual who is at risk for a disease in which they witnessed firsthand as a caregiver, there is an opportunity to promote physical and psychosocial health and wellness.

## Limitations

Study findings are limited to the perspective of participants engaging in care in an academic medical center who are largely female, well-educated and choose to engage in surveillance. Future work could target the enrollment of men to seek a more balanced sample. Additionally, outcome data were self-reported and therefore a potential risk for biased responses or socially desirable responses. Finally, we did not collect data pertaining to specific genetic mutation status, Body Mass Index (BMI), diet, or level of physical activity directly as part of this study, which would be important for future studies.

## Conclusion

In these select participants from a comprehensive cancer center, most were engaging in pancreatic cancer surveillance, and alcohol intake was moderate and tobacco intake was minimal. Modifiable factors, such as pancreatic cancer risk related distress and procedural discomfort could be intervened upon within a clinical or research context. The sample is highly motivated to engage in behavioural health intervention. It is imperative that clinical research in the era of precision medicine continues to mandate that effort be made to help individuals prevent cancer by improving health behaviours in themselves or their family. Focusing on improving overall health and wellness through healthy lifestyle behaviours (such as tobacco cessation or reduction of alcohol) is non-invasive, often inexpensive and easily accessible way to holistically promote health and potentially reduce illness in an already vulnerable population of individuals living with high risk for pancreatic cancer.
